# Sex‐Dependent Effects of Circadian Rhythm Disruptions on Novel Object Recognition Memory, Locomotion, Pain Threshold, Rearing Behavior, and BDNF and GSK‐3*β* Hippocampal Levels in Rats

**DOI:** 10.1155/bn/8686305

**Published:** 2026-07-30

**Authors:** Zeinab Dalil-Housh, Amir-Hossein Niakani, Marzieh Jalalian-Javadpour, Zahra Haghighi Poode, Leila Sepehri, Reihane Javid, Salar Vaseghi

**Affiliations:** ^1^ Department of Psychology, Ka.C., Islamic Azad University, Karaj, Iran, azad.ac.ir; ^2^ Department of Cell and Molecular Biology, Faculty of Medical Sciences, TeMS.C., Islamic Azad University, Tehran, Iran, azad.ac.ir; ^3^ Institute for Cognitive Science Studies (ICSS), Tehran, Iran, iricss.org; ^4^ Department of Psychology, Faculty of Education and Psychology, Azarbaijan Shahid Madani University, Tabriz, Iran, azaruniv.ac.ir; ^5^ Rahman Institute of Higher Education, Ramsar, Iran; ^6^ Medicinal Plants Research Center, Institute of Medicinal Plants, ACECR, Karaj, Iran, acecr.ac.ir

**Keywords:** brain-derived neurotrophic factor (BDNF), circadian rhythm, constant darkness, constant light, glycogen synthase kinase-3 beta (GSK-3*β*), sex differences

## Abstract

Evidence has shown that constant light or constant darkness significantly impairs cognitive functions and affects the mood state. The present study investigated the effect of circadian rhythm disturbances (circadian rhythm disruptions, CRDs) on locomotor activity, rearing behavior, pain threshold, novel object recognition memory, and brain‐derived neurotrophic factor (BDNF) and glycogen synthase kinase‐3 beta (GSK‐3*β*) expression levels in the hippocampus of both sexes of rats. CRD was done using a combination of constant light (48 h) and constant darkness (48 h) periods for 8 (short‐term), 16 (midterm), or 24 (long‐term) days. The results showed CRD in all protocols increased locomotor activity in males, whereas in females, only long‐term CRD increased it. Long‐term CRD increased rearing only in females, whereas in males, only midterm CRD decreased rearing. Short‐ and midterm CRD in males decreased pain threshold, whereas in females, only midterm CRD decreased pain threshold. Novel object recognition memory was impaired in both sexes following all protocols of CRD. All protocols of CRD in females downregulated BDNF, whereas in males, short‐term CRD had no effect. GSK‐3*β* was increased following long‐term CRD in both sexes. Spearman correlation test showed that BDNF function may be associated with changes in some behavioral functions in both sexes; however, GSK‐3*β* function may be associated with some behavioral changes only in females. In conclusion, we showed sex differences in the effect of CRD on locomotor activity, rearing behavior, and pain threshold. Also, GSK‐3*β* may show a sex‐dependent role in the modulation of behavioral changes induced by CRD.

## 1. Introduction

The concept of “circadian rhythms” describes intrinsic biological oscillations that recur with an approximately 24‐h periodicity and are linked to the Earth′s rotation and the alternating light/dark cycle [1]. In mammals, circadian rhythms are governed by an endogenous biological clock that requires alignment with environmental timing signals, known as “zeitgebers,” such as light, to preserve synchrony with the external environment [2, 3]. The circadian timing network consists of central pacemakers located in the suprachiasmatic nucleus (SCN) as well as peripheral clocks distributed across various tissues [1]. Accumulating evidence indicates that disruptions in circadian rhythms contribute substantially to the dysregulation of normal inflammatory rhythmicity, impaired angiogenesis, tumor development, and a range of inflammatory disorders [1]. Moreover, disturbances in circadian rhythmicity have been associated with alterations in locomotor behavior, increased depressive‐ and anxiety‐like phenotypes, and deficits in memory function [4, 5].

Previous research has demonstrated that circadian desynchronization induced by pinealectomy in rats robustly promotes depressive‐like behavior [6]. It has also been reported that alterations in the expression of circadian clock genes, including period Genes 1 and 2 (Per1 and Per2) as well as Bmal1, may contribute to the development of depressive‐like behavior in a chronic mild stress rat model [7]. Furthermore, disruption of circadian rhythms has been shown to markedly enhance both horizontal and vertical locomotor activity in rats [5]. Additionally, inversion of the circadian cycle (12 h/day for 3 consecutive days) has been found to impair memory retention in rats [8]. Another study reported that continuous light exposure produces anhedonia‐like behavior together with hyperactivity in rats [9].

Experimental rodent models of circadian rhythm disruption (CRD) have primarily focused on assessing alterations in circadian gene expression. However, behavioral abnormalities may also be associated with changes in other genes involved in neural plasticity, emotional regulation, cognitive performance, and neurogenesis. Brain‐derived neurotrophic factor (BDNF), regarded as one of the key neurotrophins within the central nervous system (CNS), has an essential role in regulating cognition and mood [10], and has been closely linked to neuropsychiatric disorders as well as behavioral dysfunctions [11–13]. Importantly, BDNF expression in the SCN exhibits a circadian pattern, characterized by higher expression during the subjective night—when light is capable of shifting circadian phase—and lower expression during the subjective day [14]. Furthermore, plasma BDNF levels display significant circadian rhythmicity in nearly 75% of women and 52% of men. Interestingly, in women, the timing of the BDNF acrophase appears independent of clock time, whereas in men, this relationship is maintained [15]. Additional experimental findings have shown that prolonged exposure to constant light significantly decreases hippocampal BDNF concentrations in rats [16]. Similarly, exposure to bright light or uninterrupted light conditions has been reported to reduce BDNF expression in the rat brain [17]. Taken together, these observations indicate that alterations in BDNF expression may represent an important mechanism underlying CRD models.

Glycogen synthase kinase‐3 beta (GSK‐3*β*) is a serine/threonine protein kinase with critical and multifaceted functions in energy metabolism, neural development, and body patterning processes [18]. Aberrant regulation of GSK‐3*β* has been linked to numerous neuropsychiatric conditions, most notably bipolar disorder [19–21]. Moreover, several investigations have emphasized the therapeutic promise of GSK‐3*β* inhibitors in the treatment of neurodegenerative and neuropsychiatric disorders [22–25]. Increased GSK‐3*β* expression has likewise been documented in various animal models exhibiting behavioral impairments [26, 27]. For instance, chronic unpredictable mild stress (CUMS), a widely accepted rodent model of depression, has been reported to enhance GSK‐3*β* activity [28, 29]. Elevated activity of GSK‐3*β* has also been associated with neuropathic pain and the emergence of mechanical allodynia, whereas pharmacological inhibition of this enzyme may provide therapeutic benefits [26, 30, 31]. Nevertheless, to date, no study has investigated GSK‐3*β* activity in a rat model of CRD.

Based on these findings, the current study was designed to evaluate the impact of CRD on behavioral performance, as well as hippocampal BDNF and GSK‐3*β* expression levels in both male and female rats.

## 2. Material and Method

### 2.1. Animals

Forty‐eight Wistar rats (female [n = 24] and male [n = 24]) (200–220 g, 8–9 weeks old) were used in the present research. All the rats were born and bred in Cognitive Neuroscience Lab, Medicinal Plants Research Center, Institute of Medicinal Plants, ACECR, Karaj, Iran. The lab had a 12:12 h light/dark cycle (lights on at 8:00 h) and a stable temperature (22°C ± 1°C). Each experimental group consisted of six female or male rats and each Plexiglas cage had six rats with free access to food and water. Also, all the experiments were done during the light hours (9:00 a.m. to 3:00 p.m.). Our experimental protocol was designed in accord with National Institutes of Health Guide for the Care and Use of Laboratory Animals [32] and in accord with ethical guidelines of ACECR. Also, The Cognitive Neuroscience Lab has Animal Ethics Certificate from ACECR which allows us to conduct animal studies.

### 2.2. CRD

In the present research, we aimed to induce CRD. We designed a combination of constant dark and constant light cycle protocol that lasted for 96 h (48 h constant light and 48 h constant darkness). CRD was designed in three protocols: (1) short‐term, which lasted for 8 days and contained 2 constant dark and constant light cycles; (2) midterm, which lasted for 16 days and contained 4 constant dark and constant light cycles; (3) long‐term, which lasted for 24 days and contained 6 constant dark and constant light cycles. All protocols started with 48 h constant light, and then, 48 h constant darkness. The open field test (as the first behavioral test) was done 15 min after removing the rats from the darkness. Experimental conditions were stringently controlled to eliminate any light exposure; specifically, light intensity was confirmed to be null during the designated dark phases.

### 2.3. Experimental Groups

**Figure 1:**
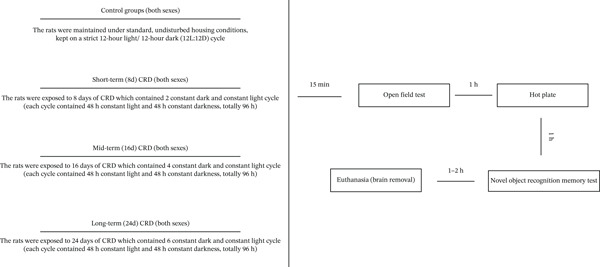
The timeline of the experiments and procedures and the experimental groups of the study.

The present study included 8 groups (*n* = 6), as follows:

• Groups 1 and 2—Control groups (both sexes): The rats in this group were maintained under standard, undisturbed housing conditions. They were kept on a strict 12‐h light/12‐h dark (12L:12D) cycle, with lights commencing at 08:00 h. Crucially, these animals were housed separately from the groups subjected to CRD.
• Groups 3 and 4—Short‐term (8 d) CRD (both sexes): The rats were exposed to 8 days of CRD which contained 2 constant dark and constant light cycles (each cycle contained 48 h constant light and 48 h constant darkness, totally 96 h).
• Groups 5 and 6—Midterm (16 d) CRD (both sexes): The rats were exposed to 16 days of CRD which contained 4 constant dark and constant light cycles (each cycle contained 48 h constant light and 48 h constant darkness, for a total of 96 h).
• Groups 7 and 8—Long‐term (24 d) CRD (both sexes): The rats were exposed to 24 days of CRD which contained 6 constant dark and constant light cycles (each cycle contained 48 h constant light and 48 h constant darkness, for a total of 96 h). The timeline of the experiments and the procedures has been provided in Figure 1.

The CRD design used in the present study represents a novel (N) approach for evaluating the effects of different circadian rhythm manipulation protocols on behavioral functions. This study introduced new light/dark phase disturbance paradigms to investigate their behavioral consequences. Previous research has commonly employed continuous light or continuous darkness conditions [33–36], or advanced light/dark phase schedules [37]. In addition, several studies have implemented prolonged CRD protocols by either shortening or extending the duration of light or dark phases over several weeks [38–40]. Other investigations have also proposed unique CRD paradigms tailored to their specific objectives [41–44]. In the current study, mixed constant light and constant darkness protocols with different durations (8, 16, and 24 days) were applied. Under these mixed conditions, rats were alternately exposed to continuous light and continuous darkness, with each phase lasting 48 h. Although combining constant light and constant darkness constituted a N experimental design, these protocols were specifically developed to examine how disruptions in the normal light/dark cycle affect locomotor activity, rearing behavior, pain threshold, N object recognition memory, and the levels of BDNF and GSK3‐*β* while allowing animals to experience both light and dark phases rather than only one condition. Therefore, the alternating exposure to constant light and constant darkness can also be considered a form of CRD, as it subjects the animals to an abnormal light/dark cycle. We used the combination of constant light and constant darkness in our recent published study [45]. However, it is important to acknowledge that the present study does not provide direct evidence confirming that the circadian rhythms were disrupted by the employed protocols. The paradigms used represent relatively new approaches to CRD, and without independent validation—such as references to prior studies or physiological indicators—it cannot be conclusively inferred that disruption occurred. As a result, the observed behavioral alterations may not necessarily reflect the effects of circadian disruption itself. To strengthen this conclusion, complementary data and measures such as home‐cage activity monitoring, corticosterone measurements, assessment of core body temperature rhythms, or evaluation of melatonin levels would be beneficial. Thus, the assumption that the CRD protocols definitively induce CRD should be regarded as tentative.

### 2.4. Open Field Test

The open field test was used to measure locomotor activity and rearing behavior in rats. During this experiment, each rat was placed in the open field test (an arena contained 16 equal‐sized squares) for 300 s. During this period, the rat was free to move through the arena. Each crossing from one square to another was considered as “one” activity. Also, the number of rearing behaviors was counted [46]. Rearing behavior consists of subject animals standing on both hind paws in a vertical upright position [47]. Rearing behavior is conventionally viewed as an index of exploratory activity and has frequently been employed to gauge anxiety levels in paradigms like the open field test and the elevated plus maze [48]. Nevertheless, a consensus regarding its valence—whether it is inherently anxiolytic or anxiogenic—is currently lacking [49]. This ambiguity is underscored by conflicting findings. Some research associates elevated rearing with increased anxiety in mice [50], whereas other studies propose that reduced rearing behavior signifies heightened anxiety [51]. Therefore, given the variability in how rearing behavior is interpreted across studies, the findings should be considered within a broader behavioral context.

### 2.5. Hot Plate

The hot plate is a behavioral test that measures the thermal pain threshold in rodents. In this test, the animal is placed on a hot plate (50°C) heated by an electric current (Tajhiz‐Gostar Omid Iranian Co, Tehran, Iran). The recording time starts from the moment the animal is placed on the hot plate and continues until the animal licks its paws. At this moment, the recording time is stopped and the pain threshold is measured. The cut‐off time is 100 s [52]. This test was done 1 h after the open field test.

### 2.6. Novel Object Recognition Test (NORT)

N object recognition memory test is a memory task that measures short‐term working memory in rodents. This test was done 1 h after the hot plate. This memory test includes three sessions: habituation, familiarization (learning), and the test. On the habituation session, the animal is habituated to the arena for 10 min (45 × 45 × 45 cm). In this session, the arena is empty and there is no special object in the arena. One hour later, the animal experiences the second phase named the familiarization phase. In this phase that lasts 10 min, two identical objects are located in opposite corners in the arena. The animal is free to explore the arena and the objects during 10 min. The last session (test) was done 24 h after the familiarization phase. During the test, one of the previous objects (in the familiarization phase) known as a familiar (F) object is replaced by a N object. Therefore, there are two objects in the test session—F and N. The animal is free to explore the arena and the two different objects for 10 min. It should be noted that F and N objects are completely different in color and shape [53]. In the test session, the time spent with N and F objects is separately recorded. The difference in the exploration time of N and F objects is calculated using the discrimination index (DI) as (time N − time F)/(time N + time F) [54]. This method was done based on our previous studies [26, 46].

### 2.7. Real‐Time PCR

After 1–2 h of the last behavioral test, the animals were exposed to CO_2_ inhalation (fill rate of 30%–70% of CO_2_ chamber) and they were decapitated, and the brain was removed from the skull. The use of CO_2_ gas has been recommended and does not lead to pain and is acceptable as described in the American Veterinary Medical Association (AVMA) guidelines [55]. Although stress is present during the euthanasia process with CO_2_, all euthanasia procedures available currently lead to an element of stress [56]. Also, CO_2_ inhalation reliably and rapidly induces loss of consciousness with minimal safety concerns for personnel [56]. In the next step, the hippocampus of each brain was extracted. Then, the total RNA was extracted from 100 mg of the hippocampus by Qiazol (Qiazol lysis reagent, United States) in a sterilized RNase‐free tube. NanoDrop ND‐100 spectrophotometer (Thermo Scientific, Waltham, Massachusetts, United States) measured the concentration and purity of RNA by the ratio of the absorbance at 260 and 280 nm (A 260/A 280). RNA was converted into complementary DNA (cDNA) by DNase I first strand synthesis system for RT‐PCR (Fermentase, Germany). Real‐time PCR reactions were done using Takara SYBR Premix Ex Taq II (Tli RNaseH Plus) (2X conc.) in a final volume of 20 *μ*L on StepOnePlus Real‐Time PCR System (Applied Biosystems). Two microliter of the synthesized cDNA was used in all reactions. The annealing temperature optimized for primer pairs was 64°C. For quantification of the target gene, the standard curve method was applied. The specificity of PCR products was verified by observing a single peak in melting curve analysis. For complementary length verification, PCR products were visualized on 2.5% agarose gel [57]. The primers were as follows: GAPDH: F ^′^AGGTCGGTGTGAACGGATTTG, R ^′^TGTAGACCATGTAGTTGAGGTCA; BDNF: F ^′^GTCCCTTCTACACTTTACCTCT, R ^′^TCTTTCACCCTTTCCACTCC; GSK3‐*β*: Forward: 5 ^′^‐GGAACTCCAACAAGGGAGCA‐3 ^′^, Reverse: 5 ^′^‐TTCGGGGTCGGAAGACCTT A‐3 ^′^.

### 2.8. Statistical Analyses

SPSS software (V.30) was used to analyze data. Two‐way ANOVA and post hoc Tukey test were used to compare groups. The effect size using partial eta squared and the observed power for each variable (sex, CRD, and sex∗CRD) were also calculated. An a priori power analysis was not conducted during the study design phase. The selected sample size (*n* = 6 per group) was based on established practices in this line of research, where similar experimental studies commonly employ comparable group sizes due to practical and methodological constraints. Although effect sizes (partial eta squared) and observed power were reported in the original manuscript, we acknowledge that these do not replace an a priori sample size justification. Therefore, a post hoc sensitivity analysis was conducted using G∗Power for a two‐way ANOVA design (sex × CRD group; 2 × 4). The analysis indicated that, with *α* = 0.05 and the current sample size (*N* = 48), the study was adequately powered to detect large effect sizes (Cohen′s *f* ≈ 0.40), whereas small to medium effects may not have been detectable. Notably, the observed effect sizes in the present study were in the medium‐to‐large range, supporting the adequacy of the sample size for detecting the reported effects. Spearman correlation test was also used to evaluate potential significant relationships between gene expression changes and behavioral alterations. Data were expressed as mean ± SD and *p* < 0.05 was considered as the level of statistical significance.

## 3. Results

### 3.1. Locomotor Activity

**Figure 2:**
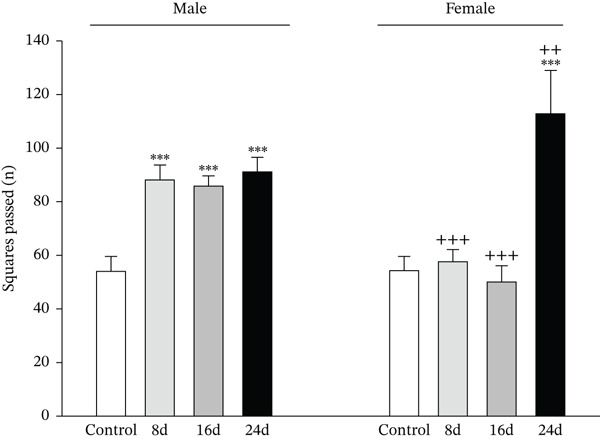
Squares passed (locomotor activity) in all experimental groups in both sexes (∗∗∗*p* < 0.001 compared with control; +++*p* < 0.001 and ++*p* < 0.01 compared with related male group; *n* = 6).

Two‐way ANOVA reports showed that the effect of sex (*F*_1,40_ = 26.00, *p* < 0.001), CRD (*F*_3,40_ = 86.31, *p* < 0.001), and sex∗CRD (*F*_3,40_ = 38.82, *p* < 0.001) was significant. The effect size for sex was as follows: partial eta squared (η_p_^2^: 0.40). Furthermore, the observed power for this effect was 1.00, confirming that the analysis had maximum capacity to detect an effect of this magnitude at the *α* = 0.05 level. The effect size for CRD was as follows: partial eta squared (η_p_^2^: 0.87). Furthermore, the observed power for this effect was 1.00, confirming that the analysis had maximum capacity to detect an effect of this magnitude at the *α* = 0.05 level. The effect size for sex∗CRD was as follows: partial eta squared (η_p_^2^: 0.74). Furthermore, the observed power for this effect was 1.00, confirming that the analysis had maximum capacity to detect an effect of this magnitude at the *α* = 0.05 level. Post hoc Tukey test revealed that locomotor activity was increased in all protocols of CRD in males (*p* < 0.001). In females, only 24 d CRD led to increased locomotor activity (*p* < 0.001). In addition, locomotor activity in 8 d and 16 d groups in males was more than respective female groups (*p* < 0.001). However, locomotor activity in 24 d group in females was more than respective male group (*p* < 0.01) (Figure 2).

### 3.2. Rearing

**Figure 3:**
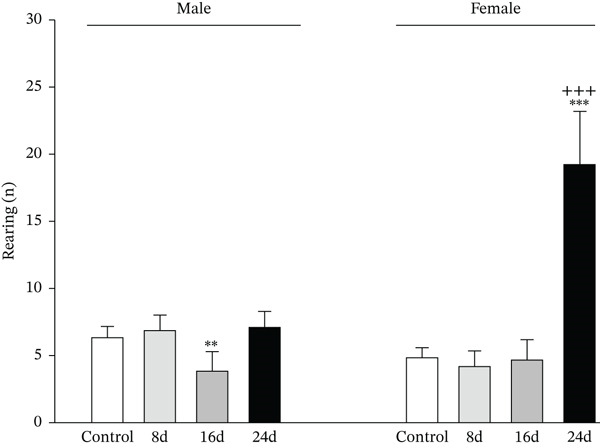
Rearing behavior in all experimental groups in both sexes (∗∗∗*p* < 0.001 and ∗∗*p* < 0.01 compared with control; +++*p* < 0.001 compared with related male group; *n* = 6).

Two‐way ANOVA reports showed that the effect of sex (*F*_1,40_ = 17.89, *p* < 0.001), CRD (*F*_3,40_ = 59.66, *p* < 0.001), and sex∗CRD (*F*_3,40_ = 42.37, *p* < 0.001) was significant. The effect size for sex was as follows: partial eta squared (η_p_^2^: 0.31). Furthermore, the observed power for this effect was 0.98, confirming that the analysis had high capacity to detect an effect of this magnitude at the *α* = 0.05 level. The effect size for CRD was as follows: partial eta squared (η_p_^2^: 0.82). Furthermore, the observed power for this effect was 1.00, confirming that the analysis had maximum capacity to detect an effect of this magnitude at the *α* = 0.05 level. The effect size for sex∗CRD was as follows: partial eta squared (η_p_^2^: 0.76). Furthermore, the observed power for this effect was 1.00, confirming that the analysis had maximum capacity to detect an effect of this magnitude at the *α* = 0.05 level. Post hoc Tukey test revealed that rearing was decreased only in 16 d group of males (*p* < 0.01). Rearing in 24 d group in females was increased (*p* < 0.001) and was more than respective male groups (*p* < 0.001) (Figure 3).

### 3.3. Pain Threshold

**Figure 4:**
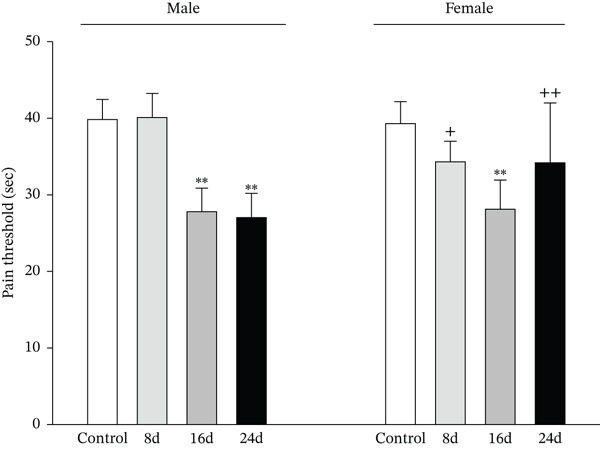
Pain threshold in all experimental groups in both sexes (∗∗*p* < 0.01 compared with control; ++*p* < 0.01 and +*p* < 0.05 compared with related male group; *n* = 6).

Two‐way ANOVA reports showed that the effect of sex (*F*_1,40_ = 0.06, *p* > 0.05) was not significant, but the effect of CRD (*F*_3,40_ = 22.51, *p* < 0.001) and sex∗CRD (*F*_3,40_ = 5.38, *p* < 0.01) was significant. The effect size for sex was as follows: partial eta aquared (η_p_^2^: 0.02). Furthermore, the observed power for this effect was 0.57. The effect size for CRD was as follows: partial eta squared (η_p_^2^: 0.63). Furthermore, the observed power for this effect was 1.00, confirming that the analysis had maximum capacity to detect an effect of this magnitude at the *α* = 0.05 level. The effect size for sex∗CRD was as follows: partial eta squared (η_p_^2^: 0.29). Furthermore, the observed power for this effect was 0.91, confirming that the analysis had high capacity to detect an effect of this magnitude at the *α* = 0.05 level. Post hoc Tukey test revealed that pain threshold was decreased in 16 d and 24 d groups of males (*p* < 0.01). In females, pain threshold was decreased only in 16 d group (*p* < 0.01). Also, pain threshold in 8 d group in females was fewer than respective male group (*p* < 0.05) and in 24 d group in females was more than respective male group (*p* < 0.01) (Figure 4).

### 3.4. NORT

**Figure 5:**
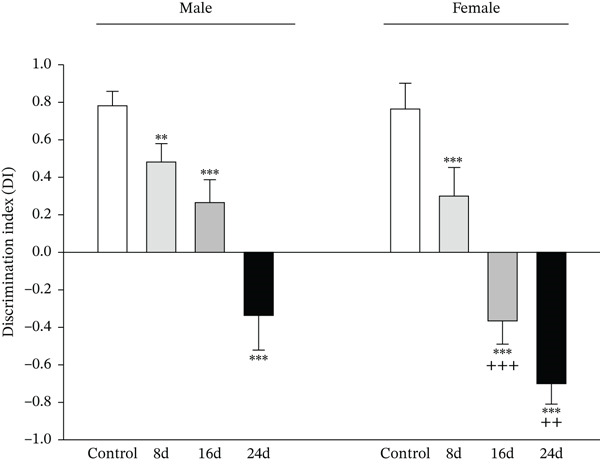
Novel object recognition memory in all experimental groups in both sexes (∗∗∗*p* < 0.001 and ∗∗*p* < 0.01 compared with control; +++*p* < 0.001 and ++*p* < 0.01 compared with related male group; *n* = 6).

Two‐way ANOVA reports showed that the effect of sex (*F*_1,40_ = 64.48, *p* < 0.001), CRD (*F*_3,40_ = 222.92, *p* < 0.001), and sex∗CRD (*F*_3,40_ = 12.50, *p* < 0.001) was significant. The effect size for sex was as follows: partial eta squared (η_p_^2^: 0.62). Furthermore, the observed power for this effect was 1.00, confirming that the analysis had maximum capacity to detect an effect of this magnitude at the *α* = 0.05 level. The effect size for CRD was as follows: partial eta squared (η_p_^2^: 0.94). Furthermore, the observed ower for this effect was 1.00, confirming that the analysis had maximum capacity to detect an effect of this magnitude at the *α* = 0.05 level. The effect size for sex∗CRD was as follows: partial eta squared (η_p_^2^: 0.49). Furthermore, the observed power for this effect was 1.00, confirming that the analysis had maximum capacity to detect an effect of this magnitude at the *α* = 0.05 level. Post hoc Tukey test revealed that N object recognition memory was impaired in all protocols of CRD in males (8 d: *p* < 0.01; 16 d and 24 d: *p* < 0.001). All protocols of CRD in females also impaired N object recognition memory (*p* < 0.001). Furthermore, impaired N object recognition memory was more significant in 16 d (*p* < 0.001) and 24 d (*p* < 0.01) groups in females compared to respective male groups (Figure 5).

### 3.5. BDNF

**Figure 6:**
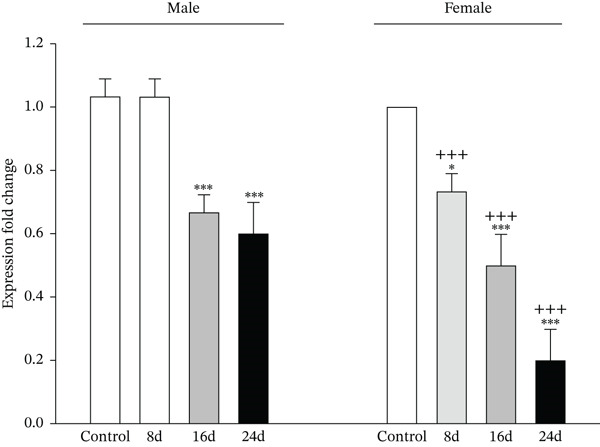
BDNF expression level in the hippocampus in all experimental groups in both sexes (∗∗∗*p* < 0.001 and ∗*p* < 0.05 compared with control; +++*p* < 0.001 compared with related male group; *n* = 3).

Two‐way ANOVA reports showed that the effect of sex (*F*_1,16_ = 56.07, *p* < 0.001), CRD (*F*_3,16_ = 87.05, *p* < 0.001), and sex∗CRD (*F*_3,16_ = 7.05, *p* < 0.01) was significant. The effect size for sex was as follows: partial eta squared (η_p_^2^: 0.78). Furthermore, the observed power for this effect was 1.00, confirming that the analysis had maximum capacity to detect an effect of this magnitude at the *α* = 0.05 level. The effect size for CRD was as follows: partial eta squared (η_p_^2^: 0.94). Furthermore, the observed power for this effect was 1.00, confirming that the analysis had maximum capacity to detect an effect of this magnitude at the *α* = 0.05 level. The effect size for sex∗CRD was as follows: partial eta squared (η_p_^2^: 0.57). Furthermore, the observed power for this effect was 0.94, confirming that the analysis had high capacity to detect an effect of this magnitude at the *α* = 0.05 level. Post hoc Tukey test revealed that BDNF expression level was decreased in 16 d and 24 d groups of males (*p* < 0.001). In females, all protocols of CRD decreased BDNF expression level (8 d: *p* < 0.05; 16 d and 24d: *p* < 0.001). Also, BDNF level was fewer in all female groups compared to respective male groups (*p* < 0.001) (Figure 6).

### 3.6. GSK‐3*β*

**Figure 7:**
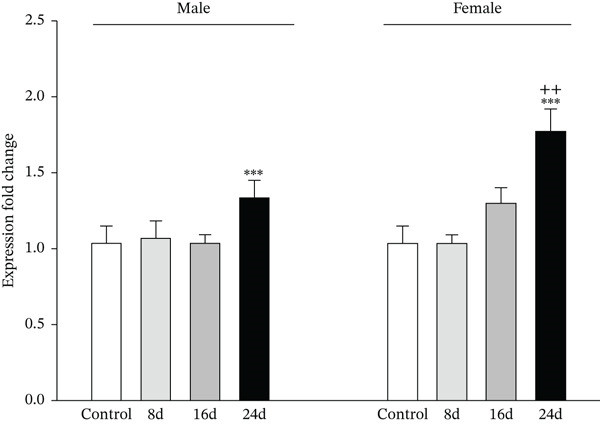
GSK‐3*β* expression level in the hippocampus in all experimental groups in both sexes (∗∗∗*p* < 0.001 compared with control; ++*p* < 0.01 compared with related male group; *n* = 3).

Two‐way ANOVA reports showed that the effect of sex (*F*_1,16_ = 14.27, p < 0.01), CRD (*F*_3,16_ = 29.81, *p* < 0.001), and sex∗CRD (*F*_3,16_ = 6.38, *p* < 0.01) was significant. The effect size for sex was as follows: partial eta squared (η_p_^2^: 0.47). Furthermore, the observed power for this effect was 0.94, confirming that the analysis had high capacity to detect an effect of this magnitude at the *α* = 0.05 level. The effect size for CRD was as follows: partial eta squared (η_p_^2^: 0.85). Furthermore, the observed power for this effect was 1.00, confirming that the analysis had maximum capacity to detect an effect of this magnitude at the *α* = 0.05 level. The effect size for sex∗CRD was as follows: partial eta squared (η_p_^2^: 0.55). Furthermore, the observed power for this effect was 0.92, confirming that the analysis had high capacity to detect an effect of this magnitude at the *α* = 0.05 level. Post hoc Tukey test revealed that GSK‐3*β* expression level was increased only in 24 d group of both sexes (*p* < 0.001). GSK‐3*β* upregulation in 24 d female group was more than respective male group (*p* < 0.01) (Figure 7).

### 3.7. Correlations

Spearman correlation test revealed a significant relationship between these factors in male rats: locomotor activity and BDNF (rho = −0.834, *p* < 0.001); N object recognition memory and BDNF (rho = 0.793, *p* < 0.001). Spearman correlation test revealed a significant relationship between these factors in female rats: rearing behavior and BDNF (rho = 0.595, *p* < 0.05); pain threshold and BDNF (rho = 0.762, *p* < 0.01); N object recognition memory and BDNF (rho = 0.879, *p* < 0.01); pain threshold and GSK‐3*β* (rho = −0.612, *p* < 0.05); N object recognition memory and GSK‐3*β* (rho = −0.687, *p* < 0.05). Thus, the results showed a positive association between BDNF levels and N object recognition memory, and a negative association between BDNF levels and locomotor activity in males. In addition, the results showed a positive association between BDNF levels and rearing behavior, between BDNF levels and pain threshold, and between BDNF levels and N object recognition memory in females. Also, there was a negative association between GSK‐3*β* levels and pain threshold, and between GSK‐3*β* levels and N object recognition memory in females (Table 1). Spearman correlation analysis revealed significant associations between behavioral measures and molecular markers. In male rats, BDNF levels were negatively correlated with locomotor activity (rho = −0.834, *p* < 0.001) and positively correlated with N object recognition memory (rho = 0.793, *p* < 0.001). In female rats, BDNF levels showed positive correlations with rearing behavior (rho = 0.595, *p* < 0.05), pain threshold (rho = 0.762, *p* < 0.01), and N object recognition memory (*r* *h* *o* = 0.879, *p* < 0.01). Additionally, GSK‐3*β* levels were negatively correlated with pain threshold (*r* *h* *o* = −0.612, *p* < 0.05) and N object recognition memory (*r* *h* *o* = −0.687, *p* < 0.05). These findings indicated that variations in BDNF and GSK‐3*β* levels were associated with changes in behavioral outcomes; however, these correlations did not imply causal relationships. Of note, the results of spearman correlation test are exploratory because all behavioral and molecular measures were obtained from the same animals and a large number of correlations were tested between multiple behavioral outcomes and two molecular markers. The observed power for all effects was 1 or near‐unity, indicating the high sensitivity and robustness of the statistical analyses. Observed power values were reported for descriptive purposes; however, they should be interpreted cautiously, particularly in studies with relatively small sample sizes. Also, given the exploratory nature of these analyses and the number of correlations performed, no formal correction for multiple comparisons was applied; therefore, the findings should be interpreted with caution.

**Table 1:** The results of spearman correlation test. It was shown that BDNF function may be associated with changes in novel object recognition memory and locomotor activity in males, and to changes in rearing behavior, pain threshold, and novel object recognition memory in females. However, GSK‐3*β* function may be associated with changes in pain threshold and novel object recognition memory only in females.

Sex	Behavioral function	Gene	Relationship
Male	Locomotor activity	BDNF	Negative
Male	Novel object recognition memory	BDNF	Positive
Female	Rearing behavior	BDNF	Positive
Female	Pain threshold	BDNF	Positive
Female	Novel object recognition memory	BDNF	Positive
Female	Pain threshold	GSK‐3*β*	Negative
Female	Novel object recognition memory	GSK‐3*β*	Negative

## 4. Discussion

As the results showed, CRD induced sex‐dependent effects on locomotor activity, rearing behavior, and pain threshold. We showed that CRD in all protocols increased locomotor activity in males, whereas in females, only long‐term CRD increased locomotor activity more than in males. Long‐term CRD increased rearing only in females, whereas in males, CRD in all protocols did not increase rearing and midterm CRD decreased rearing. Pain threshold was decreased following short‐ and midterm CRD in males, whereas in females, only midterm CRD decreased pain threshold. N object recognition memory was impaired in both sexes following all protocols of CRD. BDNF levels were decreased following all protocols of CRD in females, whereas in males, only short‐term CRD had no effect. GSK‐3*β* expression levels were increased following long‐term CRD in both sexes. Spearman correlation test showed a positive association between BDNF levels and N object recognition memory, and a negative association between BDNF levels and locomotor activity in males. It also showed a positive association between BDNF levels and rearing behavior, between BDNF levels and pain threshold, and between BDNF levels and N object recognition memory in females. Spearman correlation test also showed a negative association between GSK‐3*β* levels and pain threshold, and between GSK‐3*β* levels and N object recognition memory in females. Therefore, we showed that BDNF function may be associated with changes in N object recognition memory and locomotor activity in males, and with changes in rearing behavior, pain threshold, and N object recognition memory in females. However, we showed that GSK‐3*β* function may be associated with changes in pain threshold and N object recognition memory only in females. The observed correlations between molecular and behavioral factors must be interpreted cautiously. The correlation results presented here, derived from a limited set of groups and sample sizes, should be interpreted with circumspection. Therefore, the precise nature (positive or negative) of the relationship between the molecular and behavioral factors investigated cannot be definitively established in the present study. Further investigation with expanded sample sizes is warranted to fully elucidate these associations.

Circadian rhythms in the body are time‐tracking systems that enable our cell types and tissues to anticipate environmental alterations, thereby adapting their behavior and physiology to the time of day [58]. All these physiological and behavioral adaptations are mediated by an orchestra of molecular pacemakers in which the master clock of the SCNs sets the phase coherence between and within the peripheral clocks [59]. Evidence has shown that CRD have deleterious effects on cognition and mood. Constant light desynchronizes the molecular rhythms of neurons in the SCN [60]. It has been shown that 8 weeks of constant light potently leads to depressive‐ and anhedonic‐like behaviors in rats [61]. Induction of a short photoperiod (5 h light per day) leads to anxiety‐ and depressive‐like behaviors in rats [62]. Prenatal constant light exposure significantly induces anxiety‐like behaviors in the adult rats [63]. Three weeks of constant light also leads to increased depressive‐like behavior in the forced swim test and sucrose preference test in mice, however, anxiety‐like behavior is reduced in the open field test and elevated plus maze [64]. Other study has shown that constant light during the neonatal phase does not induce anhedonic‐like behavior, although it leads to a greater locomotor activity in rats [9]. Previous research has demonstrated that chronic constant light significantly disrupts spatial memory and affects long‐term depression (LTD) in the hippocampus of rats [65]. It has been reported that prenatal constant light has no effect on spatial learning and memory, and on recognition memory, although it increases anxiety‐like behavior [66]. Furthermore, it has been revealed that constant darkness leads to depressive‐like behaviors and induces apoptosis and neural damage in rats [67]. Constant darkness increases depressive‐like behaviors [68, 69]. In addition, it has been reported that prolonged constant darkness modifies the effect of the SCN on monoaminergic structures such as the locus coeruleus (LC) and others that are involved in modulating the mood state [67, 70]. Evidence on the effect of circadian rhythm disturbance on pain threshold is very limited. The present study used a combination of constant light and constant darkness periods. We showed that combination of constant light and constant darkness periods (CRD) for 8, 16, and 24 days increased locomotor activity in males. However, only 24‐day CRD increased locomotor activity in females. Rearing behavior was increased only following 24‐day CRD in females, whereas in males, only 16‐day CRD slightly decreased it. Rearing behavior is generally regarded as an exploratory behavior and is often interpreted as anxiety‐related behavior in both the open field test and the elevated plus maze [48]. However, its exact relationship with anxiety remains unclear, as there is no definitive evidence indicating whether rearing behavior reflects anxiolytic or anxiogenic states. Some studies have reported that increased rearing behavior is associated with elevated anxiety levels [50], whereas others have suggested that reduced rearing behavior corresponds to greater anxiety [51]. It seems that CRD has stronger deleterious effects on female rats’ performances in the open field test than males. In addition, we showed that CRD has inconsistent effects on pain threshold, showing that only midterm CRD decreased pain threshold, whereas other protocols had no effect. However, 16‐ and 24‐day CRD decreased pain threshold in males. Therefore, our data showed significant sex differences in the rats′ performance in the open field test and the hot plate. We also showed N object recognition memory deficit in both sexes following different protocols of CRD. Evidence has shown the impairment of different types of memory induced CRD. It has been shown that prenatal constant light exposure impairs short‐term memory in the adult rats [63]. Prenatal constant light induces significant impairments in spatial memory in rats [71]. It has been shown that chronic constant light significantly disrupts spatial memory in rats [65]. Other study has shown that constant light impairs spatial working memory in mice [72]. On the contrary, previous study has shown that prenatal constant light does not impair spatial learning and memory [66].

Numerous studies have documented the detrimental consequences of CRD on behavioral and cognitive functions; however, the mechanisms responsible for these effects remain incompletely understood. Circadian rhythms are strongly regulated by melatonin and its receptors. Activation of the MT2 receptor has been demonstrated to induce phase advances in the circadian rhythm of neuronal firing within SCN slice preparations [73], although a potential contribution of the MT1 receptor cannot be excluded. Available evidence indicates that the MT1 receptor is involved in melatonin‐mediated phase advances of circadian rhythms in mice [74]. Both MT1 and MT2 receptors have additionally been linked to the modulation of behaviors associated with anxiety and anhedonia [75]. More recently, melatonin has been shown to upregulate the expression of the clock genes Per1 and Per2, which play crucial roles in circadian clock resetting [76]. It has been reported that plasma melatonin concentrations are reduced in individuals with major depressive disorder (MDD), partly as a consequence of persistent neuroinflammatory activity and elevated levels of soluble pro‐inflammatory mediators [77]. Furthermore, patients with either MDD or bipolar disorder exhibit diminished responsiveness to melatonin due to a single nucleotide polymorphism located near the MTNR1A gene encoding the MT1 receptor [78]. Reduced hippocampal volume has also been observed in mood disorders, particularly depression [79], as well as in conditions involving cognitive impairment [80, 81]. Importantly, the association between the procognitive and antidepressant properties of melatonin and its regulatory effects on hippocampal neuroplasticity has been highlighted previously [79]. Melatonin may exert neuroprotective effects by counteracting NMDA receptor‐mediated glutamate excitotoxicity induced by circadian disruption, including impaired BDNF signaling and neuronal cell death [82]. It has also been shown that melatonin enhances neuroplasticity within the CNS through modulation of the glutamatergic system [79]. Melatonin produces antistress and antidepressant‐like actions, likely through interactions with several neurotransmitter systems, including the GABAergic, serotonergic, glutamatergic, and nitrergic pathways [83, 84], as well as through regulation of the hypothalamic–pituitary–adrenal (HPA) axis [85]. Melatonin has also been reported to stimulate dendritic growth and synapse formation in the hippocampus, processes that are compromised under chronic stress conditions [86]. In newly generated neurons of the dentate gyrus and differentiated hilar neurons maintained in hippocampal organotypic slice cultures, 6 h of melatonin exposure enhanced dendritic development and increased synaptic density [87]—effects that may contribute to improvements in mood and cognitive performance. The participation of both MT1 and MT2 receptors in axonal growth within human olfactory neuronal precursors, as well as in dendritic development in organotypic hippocampal slices, has likewise been documented [79]. Melatonin has additionally been shown to alleviate cognitive and memory deficits induced by sleep deprivation and ischemia‐reperfusion injury [88]. Therefore, melatonin dysfunction may represent one of the most important underlying mechanisms contributing to the effects of CRD, given its critical roles in regulating neuroplasticity, cognition, and mood [89], whereas BDNF appears to be one of the principal mediators involved in neuroplasticity regulation.

Our findings further demonstrated that CRD reduced hippocampal BDNF expression, whereas an increase in GSK‐3*β* expression was observed only after 24 days of CRD. Notably, both effects were more pronounced in female animals. BDNF is a neurotrophic factor that participates in numerous molecular, cognitive, and behavioral functions, including learning, memory formation, mood regulation, synaptic plasticity, and neurogenesis [90–93]. A growing body of evidence has documented alterations in BDNF following disturbances of circadian rhythms. Previous research has reported that chronic exposure to constant light reduces hippocampal BDNF levels in rats [16]. Another study demonstrated that bright light or continuous light exposure significantly lowers BDNF mRNA expression in the hypothalamus, cortex, and thalamus of rats [17]. Continuous light exposure has also been shown to impair hippocampal neurogenesis in mice through suppression of BDNF signaling [94]. Moreover, it has been reported that constant darkness abolishes the normal light/dark cycle‐dependent fluctuations of BDNF/TrkB expression in the hippocampus [95]. In contrast, an earlier study indicated that constant light may elevate BDNF concentrations in the cerebellum of rats [96]. The current study likewise demonstrated that all CRD protocols reduced hippocampal BDNF levels in females, whereas in males, reductions were observed following 16‐ and 24‐day CRD but not after 8 days of CRD. Spearman correlation analysis further suggested that BDNF activity may be linked to alterations in N object recognition memory and locomotor activity in males, as well as changes in rearing behavior, pain threshold, and N object recognition memory in females. Existing evidence supports a close association between circadian rhythms and BDNF. Previous work has emphasized that BDNF plays a pivotal role in regulating multiple aspects of circadian behavioral patterns and neuroendocrine functions [97]. It has been demonstrated that BDNF expression undergoes circadian oscillations in rodents, with higher expression during the dark phase in the hippocampus and cerebellum, whereas peak expression occurs during the light phase in the retina and visual cortex [98]. BDNF‐mediated signaling is considered a key component in the regulation of circadian rhythmicity [99]. In addition, direct administration of BDNF into the SCN of rats produces significant phase advances when delivered during the subjective day, a period during which the circadian clock is normally insensitive to light. Conversely, mice deficient in BDNF exhibit reduced amplitudes of light‐induced phase shifts during the subjective night [100].

On the other hand, we showed that 24‐day CRD increased GSK‐3*β* expression levels in the hippocampus of both sexes. There is no study investigating GSK‐3*β* expression changes following CRD. For the first time, we showed that long‐term CRD can increase the expression level of GSK‐3*β* in the hippocampus of both sexes, with a greater effect in females. GSK‐3*β*, together with the alternative GSK isoform, GSK‐3*α*, regulates a broad array of biological processes through Wnt signaling and several other intracellular pathways [101]. Consequently, GSK‐3*β* plays a critical role in physiological function because of its extensive interactions with multiple molecular signaling networks. GSK‐3*β* participates in numerous cellular signaling cascades, and its activity is both directly and indirectly controlled by a variety of kinases, phosphatases, and proteases [102]. Abnormal regulation of GSK‐3*β* has been linked to a wide range of pathological disorders, including diabetes and insulin resistance, neurological dysfunction, Alzheimer′s disease, schizophrenia, dopamine‐associated behavioral abnormalities, bipolar disorder, Parkinson′s disease, and cancer [103–107]. Available evidence indicates that increased activity and expression of GSK‐3*β*, rather than its inhibitory phosphorylation, are involved in the pathogenesis of behavioral impairments and mood‐related disorders [26]. For example, a previous investigation showed that aluminum exposure impairs long‐term potentiation (LTP) within the rat hippocampus, whereas administration of SB216763, a GSK‐3*β* inhibitor, markedly elevates hippocampal BDNF protein levels and increases the p‐GSK‐3*β*/GSK‐3*β* ratio, leading to improved functional outcomes [108]. Likewise, SB216763 has been reported to prevent reductions in hippocampal BDNF expression and enhance BDNF levels in the spinal dorsal horn of rats subjected to spared nerve injury, resulting in amelioration of both memory impairments and neuropathic pain [109]. Another study demonstrated that AR‐A014418, a different GSK‐3*β* inhibitor, may counteract ouabain‐induced manic‐like behaviors and locomotor abnormalities in rats [110]. Taken together, these observations suggest that GSK‐3*β* activation may represent a key mechanism underlying a wide variety of behavioral dysfunctions. The current study is the first to show that prolonged CRD results in increased hippocampal GSK‐3*β* expression, which may contribute to the emergence of behavioral disturbances. Moreover, Spearman correlation analysis revealed that GSK‐3*β* activity may be selectively related to changes in pain threshold and N object recognition memory exclusively in female animals.

## 5. Limitations and Strengths

At first, we should point out an important limitation of the present study. Although one of the strengths of the study is the use of both sexes, we did not monitor the estrous cycle of the female rats, which might have influenced their behavioral performance. The estrous cycle is known to affect pain sensitivity, mood, and BDNF function. Evidence indicates that visceral pain responses can be estrous cycle–dependent [111]. For instance, heightened visceral sensation in proestrus versus metestrus/diestrus, or the opposite pattern, has been reported [112, 113]. Moreover, several studies have demonstrated changes in BDNF levels across the estrous cycle, as well as modulation of BDNF function by estrogen [114–116]. Therefore, this limitation should be taken into account when interpreting the current findings. Second, the sample size was small, although many studies use six animals per group, but a larger sample size can make the results more valid. Third, we only assessed young adult rats (almost 8w), although the effects of circadian rhythm changes may vary at different ages and future studies could measure these effects at other ages. Fourth, we focused on assessing the gene expression levels of BDNF and GSK‐3*β* in the hippocampus. Measurement of protein levels in hippocampal homogenates (e.g., by ELISA or Western blot) was not performed due to methodological and resource limitations. We acknowledge that gene expression does not always directly reflect protein abundance or activity. Therefore, the lack of hippocampal BDNF and GSK‐3*β* protein measurements represents a limitation of our study, which should be addressed in future investigations to provide a more comprehensive understanding of the molecular mechanisms involved. Finally, as mentioned in Section 2.3, the present study does not provide direct evidence confirming that the circadian rhythms were disrupted by the employed protocols. The paradigms used represent relatively new approaches to CRD, and without independent validation—such as references to prior studies or physiological indicators—it cannot be conclusively inferred that disruption occurred. Although using the combination of constant light and constant darkness with different durations can be declared as new CRD protocols, considered as the other strengthen of the study, which was used in our recent published study with more variations [45].

## 6. Conclusion

In conclusion, we showed that the combination of constant light and constant darkness (CRD) potently affects behavioral and memory functions in both sexes of rats. We showed sex differences in the effect of CRD on locomotor activity, rearing behavior, and pain threshold. We also showed that the effect of CRD on BDNF and GSK‐3*β* expression levels in females was stronger than in males. In addition, it was shown that BDNF function may be associated with changes in N object recognition memory and locomotor activity in males and with changes in rearing behavior, pain threshold, and N object recognition memory in females. However, GSK‐3*β* function may be associated with changes in pain threshold and N object recognition memory only in females. Of note, the present study was the first study using a combination of constant light and constant darkness periods to induce CRD. Until now, the related studies have always been designed based on the constant light or the constant darkness period.

Importantly, these findings may have potential clinical implications. CRD is highly prevalent in modern societies and is commonly observed in conditions such as shift work, jet lag, sleep disorders, and various neuropsychiatric and neurodegenerative diseases [117, 118]. The present results suggest that severe circadian misalignment may contribute to impairments in cognitive functions, alterations in pain perception, and changes in behavioral activity. Notably, the observed sex‐dependent differences indicate that males and females may differentially respond to circadian disturbances, which could have important implications for understanding sex‐specific vulnerability to disorders such as depression, chronic pain conditions, and cognitive decline. Furthermore, the involvement of BDNF and GSK‐3*β* pathways provides potential mechanistic insight into how circadian disruption may influence brain function at the molecular level. Given the known roles of these pathways in synaptic plasticity, mood regulation, and neurodegeneration [119, 120], circadian disruption may represent a modifiable risk factor affecting these processes. These findings may therefore support the development of targeted interventions, including circadian‐based therapies, light exposure management, and personalized treatment strategies that take sex differences into account. However, it should be emphasized that these findings are based on an animal model, and further studies in humans are required to determine the translational relevance and clinical applicability of these results.

## Author Contributions

Z.D‐H., A‐H.N., and Z.H.P. conducted the experiments. L.S., M.J‐J., and R.J. contributed in analyzing the data, writing, drafting, and validating. S.V. designed the study, supervised the research process, validated, and revised the manuscript.

## Funding

No funding was received for this manuscript.

## Consent

The authors have nothing to report.

## Conflicts of Interest

The authors declare no conflicts of interest.

## Data Availability

The data that support the findings of this study are available on request from the corresponding author. The data are not publicly available due to privacy or ethical restrictions.
